# A multi-graph convolutional network method for Alzheimer’s disease diagnosis based on multi-frequency EEG data with dual-mode connectivity

**DOI:** 10.3389/fnins.2025.1555657

**Published:** 2025-07-02

**Authors:** Qingjie Xu, Libing An, Haiqiang Yang, Keum-Shik Hong

**Affiliations:** ^1^School of Automation, Institute for Future, Qingdao University, Qingdao, China; ^2^Shandong Key Laboratory of Industrial Control Technology, Qingdao, China; ^3^School of Pharmacy, Taishan Vocational College of Nursing, Taian, China; ^4^School of Mechanical Engineering, Pusan National University, Busan, Republic of Korea

**Keywords:** Alzheimer’s disease diagnosis, EEG, multi-graph convolutional network, dual-mode connectivity, multi-frequency analysis

## Abstract

**Objective:**

Alzheimer’s disease (AD) is mainly identified by cognitive function deterioration. Diagnosing AD at early stages poses significant challenges for both researchers and healthcare professionals due to the subtle nature of early brain changes. Currently, electroencephalography (EEG) is widely used in the study of neurodegenerative diseases. However, most existing research relies solely on functional connectivity methods to infer inter-regional brain connectivity, overlooking the importance of spatial connections. Moreover, many existing approaches fail to fully integrate multi-frequency EEG features, limiting the comprehensive understanding of dynamic brain activity across different frequency bands. This study aims to address these limitations by developing a novel graph-based deep learning model that fully utilizes both functional and structural information from multi-frequency EEG data.

**Methods:**

This paper introduces a Multi-Frequency EEG data-based Multi-Graph Convolutional Network (MF-MGCN) model for AD diagnosis. This method integrates both functional and structural connectivity to more thoroughly capture the relationships among brain regions. By extracting differential entropy (DE) features from five distinct frequency bands of EEG signals for each segment and using graph convolutional networks (GCNs) to aggregate these features, the model effectively distinguishes between AD and healthy controls (HC).

**Results:**

The outcomes show that the developed model outperforms existing methods, achieving 96.15% accuracy and 98.74% AUC in AD and HC classification.

**Conclusion:**

These findings highlight the potential of the MF-MGCN model as a clinical tool for Alzheimer’s disease diagnosis. This approach could help clinicians detect Alzheimer’s at earlier stages, enabling timely intervention and personalized treatment plans.

## 1 Introduction

Dementia is mainly identified by cognitive function deterioration, which notably affects daily bodily functions and includes several neurodegenerative conditions. Alzheimer’s disease (AD) stands as the primary form of dementia, impacting millions of individuals globally (Mayeux, 2006; Rossini et al., 2020), particularly among the elderly. AD typically progresses slowly in its early stages and worsens over time. However, clinical diagnosis of AD is challenging, as brain lesions in patients are not apparent during the initial stages (Seto et al., 2021). Current treatment focuses on artificial interventions to slow disease progression and delay its transition to the most severe stage. Diagnosing these diseases generally involves a combination of cognitive and neurological evaluations, along with neuroimaging methods like magnetic resonance imaging (MRI) (Sperling et al., 2011; Li et al., 2024). While these modalities are valuable, they are both costly and time-consuming (Babiloni et al., 2016), making them unsuitable for long-term monitoring.

Therefore, there is a need to develop a fast and cost-effective diagnostic method. Electroencephalography (EEG) is a cost-effective and accessible neuroimaging method (Li R. et al., 2019; Wang et al., 2022), which captures cumulative electrical potentials from multiple brain regions. It is ideally suited for the long-term monitoring of AD. EEG is widely applied across various fields, making it a promising alternative method. However, its potential for differential diagnosis of AD has not been fully explored. While EEG has not been extensively employed in the clinical diagnosis of AD, research points to its significant effectiveness in AD detection using EEG-based methods (Fouladi et al., 2022).

However, raw EEG data often cannot be directly applied for vigilance estimation in non-ERP experiments (Shi et al., 2013). Therefore, it is essential to derive features from EEG signals. EEG signal features are generally categorized into three main types. In the time domain, widely adopted EEG features include Hjorth parameters (Hjorth, 1970), fractal dimension (Liu and Sourina, 2013), and higher-order cross (Petrantonakis and Hadjileontiadis, 2009). Because EEG signals are represented as discrete sequences over time, time-domain features provide crucial information about the brain’s electrical patterns and its activity (Li Y. et al., 2019). Frequency-domain features analyze the frequency components of the signal, with common features including power spectral density (PSD) (Goldfischer, 1965) and differential entropy (DE) (Duan et al., 2013). Time-frequency domain features primarily include Hilbert-Huang Spectrum (Jenke et al., 2014) and wavelet transforms (Sbargoud et al., 2019). DE, in particular, can better reflect the complexity and uncertainty of signals, effectively quantifying the complexity and dynamics of EEG activity (Shi et al., 2013). This aspect is especially important for neurodegenerative diseases, as these conditions often lead to significant changes in brain activity patterns (Pievani et al., 2011; de Haan et al., 2012a). Therefore, DE features can reveal these changes and provide critical information for diagnosing neurodegenerative diseases. In this study, we focus on extracting DE features from EEG signals.

Recently, with advancements in deep learning techniques (Yoo et al., 2020; Yang and Hong, 2021; Huang et al., 2024), several researchers have combined EEG signals with deep learning techniques for related studies (Yang et al., 2023). For instance, Komolovaitė et al. (2022) employed convolutional neural networks (CNN) to analyze EEG data collected from various participants in response to visual stimuli. Amini et al. (2021) extracted time-related parameters from channels as input features for CNNs to classify AD. Chen et al. (2023) developed a classification framework integrating multi-feature fusion using CNN and Vision Transformers for AD classification. Ieracitano et al. (2019) converted channel PSD into images, employing CNNs for classification. However, while these methods utilize multi-channel data, they overlook the connectivity between electrode channels, which may limit a comprehensive understanding of brain activity.

To address these challenges, research utilizing graph-based models specifically designed for structured data has emerged (Klepl et al., 2022; Shan et al., 2022). Graph convolutional networks (GCNs) are adept at processing graph data characterized by complex topological structures (Kipf and Welling, 2017; Yang et al., 2024). They effectively extract both local and global features from nodes by aggregating information from neighboring nodes (Gilmer et al., 2017). GCNs show promise across various fields, including emotion recognition and the identification of neurodegenerative conditions. For instance, Adebisi et al. (2024) introduced a GCN framework that utilizes EEG signals and employs a phase lag index to create a connectivity network for Alzheimer’s disease (AD) classification. Additionally, Leng et al. (2023) developed a GCN framework that captures directional characteristics from brain networks and analyzes the connectivity of EEG data for AD classification.

Although many researchers have proposed EEG signal processing frameworks based on GCN, these methods have indeed enhanced the classification and analysis performance of EEG signals to some extent. However, most studies overlook spatial connectivity between channels, which can reveal synchronized activity patterns between different brain regions, thus providing valuable structural information (Pons et al., 2010). Most existing research relies solely on functional connectivity methods to infer inter-regional brain connectivity, failing to fully capture the complex interactions within EEG signals. Additionally, many approaches do not adequately integrate multi-frequency EEG features, limiting the comprehensive understanding of the dynamic changes in brain activity across different frequency bands.

**FIGURE 1:**
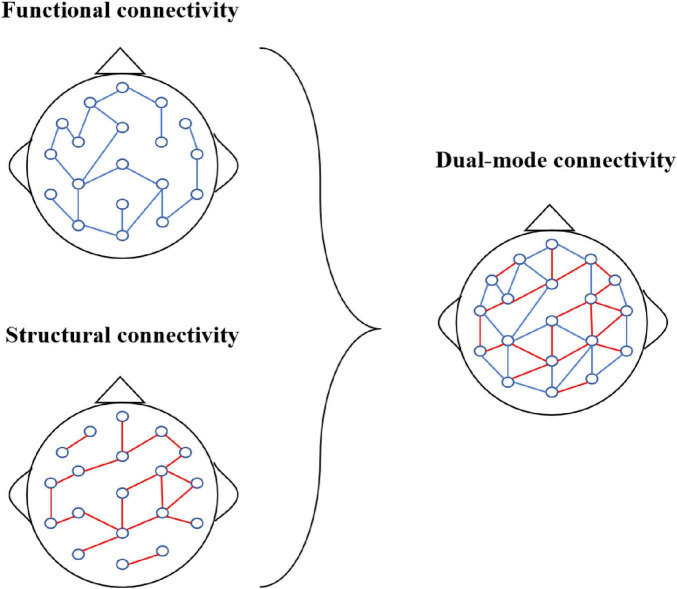
Dual-mode connectivity.

Therefore, this paper introduces a Multi-Frequency EEG data-based Multi-Graph Convolutional Network (MF-MGCN) model for AD diagnosis. The neuropathological features of AD include the deposition of β-amyloid plaques, tau protein tangles leading to synaptic dysfunction, and widespread degeneration of brain functional networks (Braak and Braak, 1991). The model uses a multi-graph structure to separately model the functional network connectivity of five different frequency bands, capturing the degradation of linear interactions between brain regions in AD patients and the abnormal connectivity patterns across different frequency bands in these patients. This model combines Pearson correlation coefficients with spatial connectivity to enhance the ability to capture relationships between nodes in EEG signals. The Pearson correlation measures the functional link intensity among electrodes in various brain regions, reflecting their linear correlation in EEG activity. In contrast, spatial connectivity focuses on the structural connections between various brain regions in physical space. The schematic diagram of the combination of functional connectivity and structural connectivity is shown in Figure 1. This combination allows the framework to grasp functional associations and structural characteristics of brain activity, thereby improving its ability to analyze EEG data comprehensively.

The main contributions of this paper are as follows:

• The framework considers the influence of various EEG frequency ranges on AD diagnosis. Hence, we introduced a multi-graph convolutional architecture in the methodology, and DE features from five distinct frequency bands are then extracted from each segment.
• Our model employs a 90% sliding window overlap, utilizing GCN to aggregate feature information in both the time-frequency and spatial dimensions. By integrating functional connectivity (the correlation of DE features between channels) and structural connectivity (the spatial relationships between channels), this combination offers richer information compared to single connectivity methods, forming a dual-mode connectivity approach that enhances the overall analysis.
• We performed thorough evaluations using the dataset (ds004504) from the OpenNeuro repository to assess our model, and the findings indicate that the approach delivers strong classification results.

This paper will be structured around the following key points: Section 2 outlines the methodology, Section 3 describes the experiments, Section 4 showcases the results, followed by the discussion in Section 5. Lastly, Section 6 provides conclusions and suggests future directions.

## 2 Materials and methods

**FIGURE 2:**
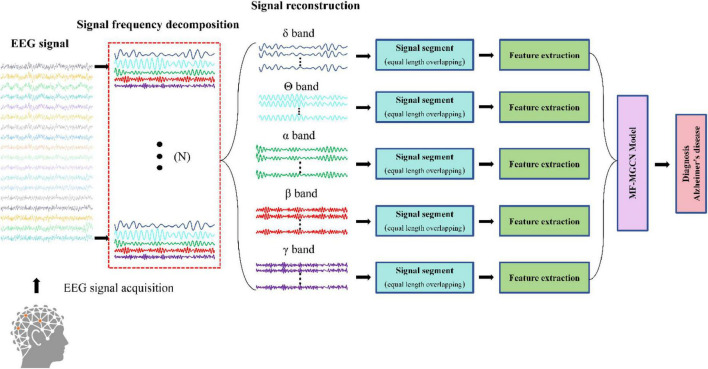
Procedure for Alzheimer’s disease diagnosis.

Figure 2 illustrates the process of Alzheimer’s disease diagnosis. Initially, EEG signals are collected and then undergo frequency band decomposition. The EEG signals within the same frequency band are subsequently restructured. Afterward, the restructured signals are segmented, and relevant features are extracted from each segment. These features are then input into the MF-MGCN model to perform the diagnosis of Alzheimer’s disease. The graph structure design of GCN can simulate the topological properties of the human brain network. The nodes represent the positions of the *N* EEG electrode locations, with node features being the DE values that reflect the complexity of the EEG signals. The edges (connection weights) are calculated based on the Pearson correlation coefficient, which quantifies the functional connectivity strength between electrodes and the degradation of connectivity in AD patients. Previous studies have shown that, compared to healthy controls (HC), the brain functional networks of AD patients exhibit degeneration (Braak and Braak, 1991). The graph convolution operation in GCN can capture these topological changes, making the GCN architecture more effective in capturing the differences in EEG signals between HC and AD patients.

**FIGURE 3:**
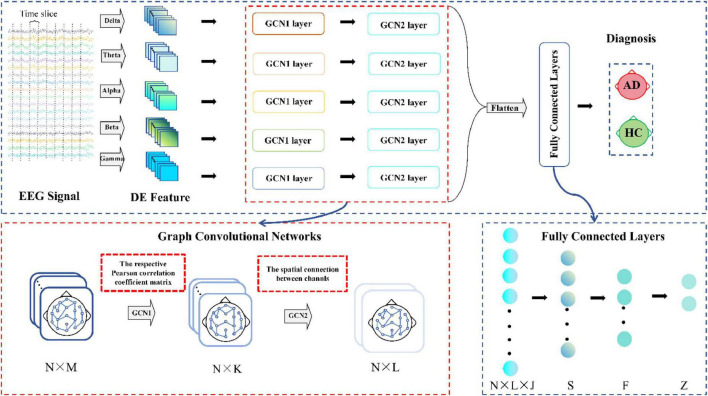
Architecture of the MF-MGCN model. *N* refers to the EEG signal channels, while *M* and *L* denote the input and output sizes for the GCN layers, respectively, and *K* represents the count of hidden units within the GCN layer. *N* × *L* × *J* and *Z* represent the input and output sizes of the fully connected layers, *J* represents the count of frequency bands, while *S*, *F* indicate hidden layer units.

Figure 3 illustrates the architecture of the MF-MGCN model. First, EEG data is segmented using a windowing method, dividing it into overlapping T-second segments. DE features from five distinct frequency bands are then extracted from each segment. The architecture consists of two GCN layers. Each electrode channel from the EEG signal is considered a node in the GCN structure. Pearson correlation coefficients among the DE features from the *N* electrode channels are calculated to construct the adjacency matrix for the first GCN layer, while the spatial connectivity between the N electrode channels serves as the adjacency matrix for the second GCN layer. The model then sets up three fully connected layers. We extracted the DE features from various frequency bands, and after convolutional aggregation through the GCN layers, the features from all bands are combined and input into the fully connected layers to ultimately classify AD and HC.

**FIGURE 4:**
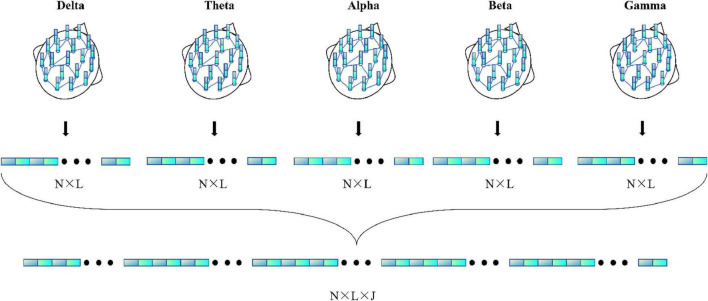
Detailed process of the flattening.

Figure 4 illustrates the detailed process of flattening in the MF-MGCN framework. In this process, features from different frequency bands have already been aggregated using GCN, then fused together, and finally, the fused features are input into the fully connected layer for further processing.

### 2.1 Feature extraction techniques

DE effectively captures signal variability and uncertainty, providing numerical measures of dynamics in EEG activity (Shi et al., 2013). In this study, DE features are derived from each segment of the EEG data, and the mathematical expression of DE is defined as follows:


(1) $$D\,E\,\left(x\right)=-\int_{-\infty }^{+\infty }s\,\left(x\right)\,\log_{2}\left(s\,\left(x\right)\right)\,dx$$

where *x* represents the time variable of the EEG data, *s*(*x*) is the probability density function of *x*. The EEG time series is approximately Gaussian distributed, with the signal’s mean denoted as μ and variance as σ^2^.


(2) $$s(x)=\frac{1}{\sqrt[{}]{2\pi \sigma_{i}{}^{2}}}\exp(-\frac{{\left(x-\mu_{i}\right)}^{2}}{2\sigma_{i}{}^{2}})\;i\in \{\delta ,\theta ,\alpha ,\beta ,\gamma \}$$

(3) $$\begin{array}{c} DE_{i}(x)=\int_{-\infty }^{+\infty }\frac{1}{\sqrt{2\pi \sigma_{i}{}^{2}}}\exp(-\frac{\left(x-\mu i\right)}{2\sigma_{i}^{2}})\log_{2}\\ (\frac{1}{\sqrt{2\pi \sigma_{i}{}^{2}}}\exp(-\frac{{\left(x-\mu_{i}\right)}^{2}}{2\sigma_{i}^{2}}))dx\\ =\frac{1}{2}\log_{2}(2\pi e\sigma_{i}^{2})\;i\in \left\{\delta ,\theta ,\alpha ,\beta ,\gamma \right\} \end{array}$$

where *DE*_*i*_(*x*) represents the DE of the *i*-th frequency band, μ_*i*_ and $\sigma_{i}^{2}$ represent the mean and variance of the signal in the *i*-th frequency band, *x*∼(μ_*i*_,σ_*i*_^2^), e and π are constants. In this study, DE features are drawn from five core EEG frequency ranges: δ-band (0.5 ∼ 4 Hz), θ-band (4 ∼ 8 Hz), α-band (8 ∼ 13 Hz), β-band (13 ∼ 25 Hz), and γ-band (25 ∼ 45 Hz).

### 2.2 Graph convolution network

#### 2.2.1 General representation of graph

A graph is defined as *G* = {*V*,*E*,*A*}, with *V* indicating the node set (|*V*| = *N*|), *E* denoting the connections or edges linking the nodes, and *A* ∈ *R*^*N*×*N*^ being the adjacency matrix showing the strength of connections between nodes.

#### 2.2.2 Node features and graph learning

Each electrode channel from the EEG signal is considered a node in the GCN structure, and the node features are defined as the DE calculated from an EEG data segment of duration *T*. Thus, the input is a node feature matrix *X* ∈ *R*^*N*×*M*^, The node feature matrix is normalized before being fed into the GCN. The Pearson correlation coefficient matrix between each pair of node features is computed as the adjacency matrix for the first GCN layer, while the spatial connectivity between the *N* electrode channels serves as the adjacency matrix for the second GCN layer. The adjacency matrix is denoted as *A* ∈ *R*^*N*×*N*^, where *N* = 19 indicates the quantity of EEG electrode channels.

#### 2.2.3 Multi-graph convolutional network section

Within the GCN part, we introduce a multi-graph convolutional network that takes into account five EEG frequency bands. Features from each frequency band are treated as distinct graph feature matrices. The model is designed with two graph convolutional layers: the first layer’s adjacency matrix relies on Pearson correlation coefficients, whereas the second layer’s adjacency matrix depends on spatial connectivity between nodes. This setup combines functional and structural connectivity, leveraging multiple types of relational information between nodes.

The multi-graph for the first convolutional layer is defined as follows:


(4) $$X_{i}^{1}=\sigma \,\left({\hat{D}}_{1\,i}^{-\frac{1}{2}}\,{\hat{A}}_{i}^{1}\,{\hat{D}}_{1\,i}^{-\frac{1}{2}}\,X_{i}^{0}\,W_{i}^{0}\right)\,\begin{array}{cc} & i\in \left\{\delta ,\theta ,\alpha ,\beta ,\gamma \right\} \end{array}$$

where $X_{i}^{0}$ represents the input feature matrix corresponding to the *i*-th frequency band, and $X_{i}^{1}$ stands for the output of the first layer for that frequency band. Here, $W_{i}^{0}$ is the learnable parameter matrix associated with the input layer, ${\hat{D}}_{1\,i}$ denotes the degree matrix of the graph, σ⋅ denotes the activation function, and ${\hat{A}}_{i}^{1}$ represents the adjacency matrix for the first layer in the *i*-th frequency band. Each element $a_{m\,n}^{1}$ in ${\hat{A}}_{i}^{1}$ is derived from the Pearson correlation among the related feature nodes for that frequency band.

The elements $a_{m\,n}^{1}$ of the adjacency matrix ${\hat{A}}_{i}^{1}$ in the first convolutional layer are defined as follows:


(5) am⁢n1=ρxm⁢y=n∑(xm-x¯)⁢(yn-y¯)∑(xm-x¯)2⁢(yn-y¯)2

m,n∈{F⁢p⁢1,F⁢7,F⁢3⁢⋯⁢⋯⁢O⁢2}

ρ_*x*_*m*_*y*_*n*__ represents the Pearson correlation coefficient between *x*_*m*_ and *y*_*n*_, where *x*_*m*_ and *y*_*n*_ indicate the respective feature values for nodes *m* and *n*. $\bar{x}$ and $\bar{y}$ represent the mean feature values for nodes *m* and *n*.

The multi-graph for the second convolutional layer is defined as follows:


(6) $$X_{i}^{2}=\sigma \,\left({\hat{D}}_{2\,i}^{-\frac{1}{2}}\,{\hat{A}}_{i}^{2}\,{\hat{D}}_{2\,i}^{-\frac{1}{2}}\,X_{i}^{1}\,W_{i}^{1}\right)\,\begin{array}{cc} & i\in \left\{\delta ,\theta ,\alpha ,\beta ,\gamma \right\} \end{array}$$

where $X_{i}^{1}$ represents the input to the second layer for the *i*-th frequency band, while $X_{i}^{2}$ stands for the output of the second layer for the same band. $W_{i}^{1}$ refers to the learnable parameter matrix associated with the second layer, ${\hat{D}}_{2\,i}$ denotes the degree matrix of the graph, σ⋅ denotes the activation function, and ${\hat{A}}_{i}^{2}$ represents the adjacency matrix for the second layer in the *i*-th frequency band.

Based on the International 10-20 system electrode layout, the 19 electrodes are assigned to different anatomical brain regions. Frontal Lobe: Fp1, Fp2, F7, F3, Fz, F4, F8; Central Region: C3, Cz, C4; Parietal Lobe: P3, Pz, P4; Temporal Lobe: T3, T4, T5, T6; Occipital Lobe: O1, O2. If two electrodes belong to the same brain region, the corresponding value in the structural adjacency matrix is 1; otherwise, it is 0.

The elements $a_{m\,n}^{2}$ of the adjacency matrix ${\hat{A}}_{i}^{2}$ in the second convolutional layer are defined as follows:


(7) $$a_{m\,n}^{2}=\left\{ \begin{array}{cc} 1, & \text{if }\,R\,\left(m\right)=R\,\left(n\right)\\ 0, & \text{otherwise} \end{array}\right.\,\begin{array}{ccc} & & m,n\in \left\{F\,p\,1,F\,7,F\,3\,\cdots \,\cdots \,O\,2\right\} \end{array}$$

where *R*(*m*) and *R*(*n*) represent the brain regions of channels *m* and *n*, respectively. If *R*(*m*) = *R*(*n*), it indicates that the channels belong to the same brain region.

## 3 Experiment

### 3.1 Dataset

The dataset employed in this research was obtained from the OpenNeuro repository (ds004504) (Miltiadous et al., 2023). It consists of EEG recordings from 65 participants, including 36 AD patients and 29 HC subjects. The cognitive abilities of these participants varied significantly, and their cognitive function was rigorously assessed using the MMSE (Kurlowicz and Wallace, 1999). Participants with AD typically presented with lower MMSE scores, reflecting greater levels of cognitive deterioration, while the HC group generally scored higher, representing normal cognitive functioning. All participants were in a quiet, undisturbed environment during EEG data collection, ensuring they were in an eyes-closed resting state (without external task stimulation) and remained awake and relaxed.

EEG recordings followed the standard 10-20 international system, with a configuration of 19 scalp electrodes placed at specific locations. The recording durations differed across the different groups. For the AD group, the average session lasted approximately 13.5 min, the range is from 5.1 to 21.3 min. In comparison, the HC group had an average recording time of 13.8 min, with a shorter range, from 12.5 to 16.5 min. To ensure consistency and comparability across participants, a 5-min segment was selected from each participant’s EEG recordings for further analysis. By unifying the data length, we ensured that the subsequent feature extraction and analysis processes could be applied uniformly across both groups.

### 3.2 Data preprocessing

The preprocessing of the EEG data was performed by the original data collectors (Miltiadous et al., 2023), involving the removal of irrelevant noise, reduction of artifacts, and ensuring clear and reliable data. These preprocessing steps not only enhanced the signal quality but also significantly improved the accuracy of the analysis results. In our study, we further processed the EEG data by segmenting it into overlapping T-second windows using a sliding window technique. The DE features were derived from every frequency band within these EEG segments. Because the participant group was relatively limited, we applied data augmentation by using a 90% overlap in the sliding windows to increase the sample size. This method expanded the dataset tenfold, growing the original 65 samples to 650, including 360 AD samples and 290 HC samples.

### 3.3 Evaluation indices

We evaluated the MF-MGCN model using multiple indices. The classification accuracy is defined as follows:


(8) $$A\,c\,c=\frac{T\,P+T\,N}{T\,P+T\,N+F\,P+F\,N}.$$

Precision is defined as:


(9) $$P\,r\,e=\frac{T\,P}{T\,P+F\,P}.$$

The calculation method for Recall is defined as:


(10) $$R\,e\,c=\frac{T\,P}{T\,P+F\,N}.$$

The calculation method for F1-score is defined as:


(11) $$F\,1-s\,c\,o\,r\,e=2^{*}\,\frac{P\,r\,e^{*}\,R\,e\,c}{P\,r\,e+R\,e\,c}.$$

ROC-AUC functions as a metric that evaluates overall efficacy. Mathematically, the ROC can be expressed as:


(12) $$T\,P\,R=\frac{T\,P}{T\,P+F\,N},$$

(13) $$F\,P\,R=\frac{F\,P}{F\,P+T\,N},$$

where TP, TN, FP, and FN represent True Positive, True Negative, False Positive, and False Negative, respectively.

### 3.4 Hyperparameters

The proposed MF-MGCN model incorporates both convolutional and fully connected layers and is implemented using Pytorch. We conducted various experiments, exploring a range of combinations and layer depths for the convolutional layers, as well as for the fully connected layers, the EEG segment duration (*T*), and other hyperparameters, to determine the most effective architecture for optimal performance. According to the literature (Adebisi et al., 2024), a segment length of 10 seconds for EEG signals is the optimal value. The total parameter count for MF-MGCN is 29,332, which is comparable to other EEG deep learning models (Klepl et al., 2022). The original data was expanded to 650 samples (360 AD ++ 290 HC) using a 90% overlapping sliding window, which aligns with the common data augmentation method for small sample EEG studies (Roy et al., 2019). The configuration parameters of the MF-MGCN framework are presented in Table 1.

**TABLE 1:** Configuration parameters of the MF-MGCN model.

Parameters for the MF-MGCN model	Values
Model learning rate λ	0.001
Model batch size	10
Model training Epoch	200
Model loss function	Cross entropy
GCN activation function type	ReLU
EEG signal segment length *T*	10 s
Number of frequency bands *J*	5
Dimensions *M* and *L* of input and output features of the GCN layer	32 and 2
The node count *N* within the graph	19
Number of model parameters	29,332

## 4 Results

### 4.1 Implementation details

Due to the process of data augmentation, several samples were created for each participant. To avoid data leakage, data splitting was performed at the participant level. Samples generated from the same participant were treated as a single group. When extracting samples for the training or testing sets, they were selected by group (participants), ensuring that the training and testing sets were completely separated by participant IDs. All augmented samples from the same participant belong exclusively to either the training set or the testing set. We randomly selected 80% of these groups for training purposes, resulting in 520 samples, and the other 20% were allocated to the test set, including 130 samples. This procedure ensured that no data from a single participant overlapped between the training and test sets. In order to reduce the risk of overfitting, a 5-fold cross-validation method was utilized during the training process.

### 4.2 Model performance

**FIGURE 5:**
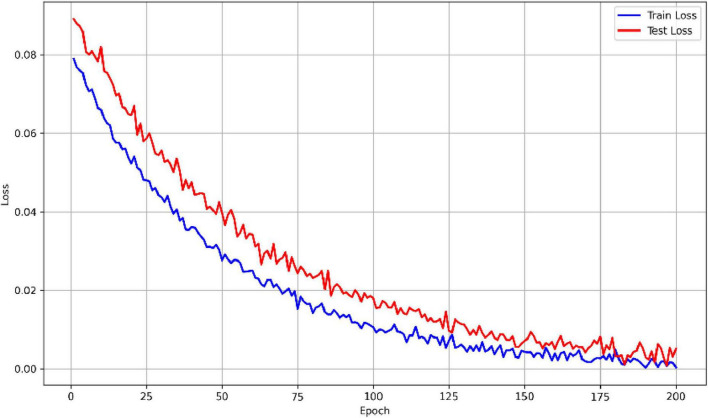
Comparison of training and testing loss curves.

Using the aforementioned methods, the outcomes of the MF-MGCN model’s classification for AD and HC are presented in Table 2. It demonstrates that the MF-MGCN achieved a classification accuracy of 96.15% and an AUC of 98.74% among the 65 subjects, showcasing excellent classification performance. The training and testing loss curves are shown in Figure 5. As illustrated in the figure, both the training and testing losses exhibit a smooth downward trend and remain closely aligned throughout the training process, with no significant divergence or upward rebound in testing loss. This indicates that the model did not experience noticeable overfitting during training.

**TABLE 2:** Classification results of different models for AD and HC.

Model	Accuracy (%)	Precision (%)	Recall (%)	F1-score (%)	AUC (%)
XGBoost	83.19	80.35	83.23	81.76	91.62
RF	89.10	88.41	87.38	87.89	95.81
ET	90.24	89.72	88.59	89.15	96.64
KNN	94.72	94.66	93.42	94.04	96.64
LCADNet	93.14	96.50	95.00	95.76	–
CNN	79.45	76.32	76.06	77.60	–
CNN-ViT	87.33	–	84.56	–	88.19
MF-MGCNI	91.54	92.98	88.33	90.60	96.98
MF-MGCNII	93.85	94.83	91.67	93.22	97.57
MF-MGCNIII	93.79	93.43	94.64	94.03	97.58
MF-MGCN	**96.15**	**97.67**	**98.33**	**98.00**	**98.74**

We conducted a comparative evaluation using the same dataset, comparing the classification results of MF-MGCN with other models, including XGBoost (Lal et al., 2024), Random Forest (RF) (Lal et al., 2024), Extra Trees (ET) (Lal et al., 2024), K-Nearest Neighbors (KNN) (Lal et al., 2024), LCADNet (Kachare et al., 2024), CNN (Stefanou et al., 2025), and CNN-ViT (Chen et al., 2023), as well as three variants of the MF-MGCN model: MF-MGCNI, MF-MGCNII and MF-MGCNIII. MF-MGCNI uses a single layer of graph convolution, with the adjacency matrix calculated using Pearson correlation coefficients. MF-MGCNII employs two layers of graph convolution, where each layer’s adjacency matrix is also calculated using Pearson correlation coefficients. MF-MGCNIII replaces the DE features with PSD features. The comparative outcomes are presented in Table 2. It is evident that the MF-MGCN outperformed the other models.

The performance of MF-MGCN exceeded that of its variants, MF-MGCNI and MF-MGCNII, indicating the effectiveness of incorporating spatial connectivity as the adjacency matrix in the second layer. After replacing the DE features with PSD features, the performance of model MF-MGCNIII shows a 2.36% decrease in accuracy (from 96.15 to 93.79%). From these results, it is clear that the combination of the graph structure architecture and DE features is irreplaceable and has a significant contribution to the classification accuracy. Based on the degree of accuracy reduction, it can be concluded that the graph structure architecture contributes more to the classification accuracy than the DE features. Although MF-MGCNI and MF-MGCNII did not outperform KNN in some metrics, they were still superior to XGBoost, RF, and ET. Among the traditional models, KNN performed the best, while RF and ET produced very similar results. Overall, our model demonstrated effectiveness in classifying AD and HC. It should be noted that LCADNet and CNN lack the AUC metric, and CNN-ViT is missing Precision and F1-score metrics, as the relevant literature did not provide these results.

We also validated the significance of the performance differences between MF-MGCN and the baseline models using the following methods: For the F1-score, we applied the Wilcoxon signed-rank test and quantified the effect size using Cohen’s *d*; for the AUC, we conducted significance analysis using the Delong test and reflected the actual improvement through the AUC difference. The statistical analysis results are shown in Table 3. With the exception of GCN, the *p*-values for both F1-score and AUC in the comparisons between MF-MGCN and the baseline models were significantly lower than the statistical significance level (α = 0.05), indicating statistically significant differences. To control for the potential Type I error inflation due to multiple hypothesis testing, we applied False Discovery Rate (FDR) correction using the Benjamini-Hochberg procedure to the original *p*-values reported in Table 3. Although some FDR-adjusted *p*-values slightly exceed the conventional significance threshold of 0.05, the overall trends remain statistically suggestive. Combined with large effect sizes (Cohen’s *d* > 0.8) and AUC differences above 2%, the results still support a meaningful performance improvement of MF-MGCN over baseline models.

**TABLE 3:** Statistical analysis of the performance difference between MF-MGCN and baseline models.

Model	F1-score difference *P*-value	F1-score FDR-adjusted p	AUC difference *p*-value	AUC FDR-adjusted p	F1-score effect size	AUC Difference (%)
SVM	0.0215	0.0549	0.0182	0.0429	2.4381	4.7004
CNN	0.0327	0.0549	0.0284	0.0429	1.8204	5.2955
GCN	0.0631	0.0631	0.0429	0.0429	0.8513	2.3511
GAT	0.0412	0.0549	0.0365	0.0429	2.1261	4.3011

*p* < 0.05 indicates a statistically significant difference, Cohen’s *d* > 0.8 means a large effect size, 0.5-0.8 represents a medium effect size, and < 0.5 implies a small effect size, AUC difference > 2.0% can be considered a meaningful improvement in practical performance.

We also performed a noise ceiling analysis on the accuracy to assess the theoretical performance limit that MF-MGCN can achieve. Using the bootstrap method, we resampled 100 times to calculate the theoretical maximum classification accuracy. The analysis results indicate that the noise ceiling is 97.8% (95% CI: 96.8–98.2%), and the accuracy of MF-MGCN is 96.15%, with only a 1.65% difference from the theoretical limit. This suggests that the model’s performance is close to the theoretical limit of the data and is minimally affected by data noise.

Finally, we have compared MF-MGCN with other DNN models, and the comparison results are shown in Table 4. As indicated in Table 4, MF-MGCN outperforms similar studies, even with comparable or smaller sample sizes.

**TABLE 4:** Comparison of MF-MGCN model performance with similar AD diagnostic models.

Study (year)	Model type	Data modality	Sample size	Accuracy(%)
Geng et al. (2022)	GRU	EEG	AD: 20 HC: 20	93.46
Shan et al. (2022)	ST-GCN	EEG	AD: 19 HC: 20	92.30
Chen et al. (2023)	DBN	EEG	AD: 36 HC: 29	87.33
Klepl et al. (2023)	AGGCN	EEG	AD: 20 HC: 20	90.50
Lin et al. (2020)	ELM	MRI+PET	AD: 102 HC: 200	84.7
Abrol et al. (2020)	ResNet	MRI	AD: 157 HC: 237	89.3
Suk et al. (2016)	Deep sparse multi-task learning	MRI	AD: 51 HC: 52	90.36
This study	MF-MGCN	EEG	AD: 36 HC: 29	96.15

## 5 Discussion

### 5.1 Differences in DE features across different frequency bands between AD and HC

**FIGURE 6:**
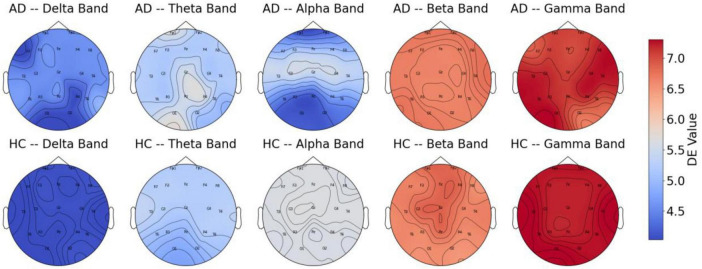
DE features of AD and HC across different frequency bands.

In the present research, we performed a comparison of DE features across different frequency bands between AD patients and HC, selecting samples from both groups, with results presented in Figure 6. The figure reveals significant differences in DE values across various frequency bands. The findings show that AD patients exhibit higher DE values than HC in the Delta and Theta bands, potentially linked to abnormal synchronization of brain neurons and cognitive decline. The increased power in the Delta and Theta bands in AD patients has been widely reported (Jeong, 2004). The elevation of DE values in these bands in the AD group in our study corroborates the findings of increased power reported by Jeong (2004). In contrast, in the mid-to-high frequency bands, the DE values of AD patients are lower than those of HC, with the most pronounced difference observed in the Alpha band. This indicates a marked reduction in brain activity related to cognition, attention, and memory functions in AD patients, the reduced complexity in the Alpha band in AD patients is directly related to the degeneration of the DMN functional connectivity (de Haan et al., 2012b). whereas healthy individuals exhibit stronger brain activity in these bands.

These findings further support the characteristic neurodegenerative pattern of AD, where low-frequency activity is enhanced, and high-frequency activity is diminished. This provides strong evidence for using EEG signals to detect Alzheimer’s disease. Moreover, the results suggest that DE features across different frequency bands can effectively distinguish between AD patients and HC.

### 5.2 Analysis of differences in functional connectivity strength between AD and HC

**FIGURE 7:**
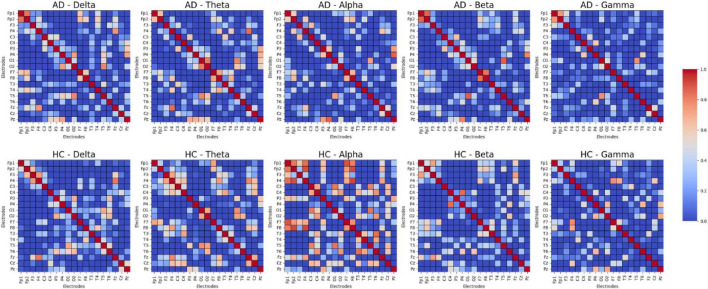
Functional connectivity strength between AD and HC.

In the present research, we performed a comparison of functional connectivity strength across different frequency bands between AD and HC, selecting a subset of samples. The results, as shown in Figure 7, reveal notable distinctions between the two cohorts across the various frequency ranges. The findings indicate that the HC group exhibits significantly stronger functional connectivity in most frequency bands, especially within the Theta, Alpha, and Gamma frequency ranges, suggesting that healthy individuals have stronger brain region connectivity in these bands. In contrast, the AD group shows a general reduction in functional connectivity across these bands, especially in those closely associated with cognition and memory. These observations highlight the diminished functional connectivity in AD patients across multiple frequency bands, reflecting substantial changes in brain activity patterns and further supporting its characterization as a neurodegenerative disease.

### 5.3 Relationship between resting-state EEG biomarkers and DE features

We systematically explain the relationship between DE features and classic resting-state EEG biomarkers. The DE features used in the model are consistent with EEG abnormal patterns related to AD at multiple levels, offering good biological interpretability.

PSD Level: DE is the logarithmic energy integral of the PSD, and its trend is highly consistent with classic PSD features. Previous studies have shown that AD patients have increased PSD in the Theta band and decreased PSD in the Alpha band (Jeong, 2004). The DE features exhibit the same trends in these bands, showing greater sensitivity to subtle frequency band fluctuations and more detailed capture of the nonlinear fluctuations in frequency domain features (Shi et al., 2013).

Functional Connectivity Level: We used DE features to compute the Pearson correlation between channels, constructing a functional connectivity network that is input to the GCN model. Figure 7 shows that the functional connectivity strength in the AD group is lower than in the HC group, which is consistent with existing research. Compared to other features, DE is more sensitive to pathological connection weakening when constructing the connectivity matrix, making it a more effective indicator of the weakened functional connections between brain regions.

Signal Complexity Level: DE is essentially a frequency-domain quantification of signal complexity. As shown in Figure 5, the DE value in the Alpha band significantly decreases in AD patients, reflecting a reduction in the complexity of EEG signals in that band for AD patients.

### 5.4 Effect of hyperparameters on model performance

**FIGURE 8:**
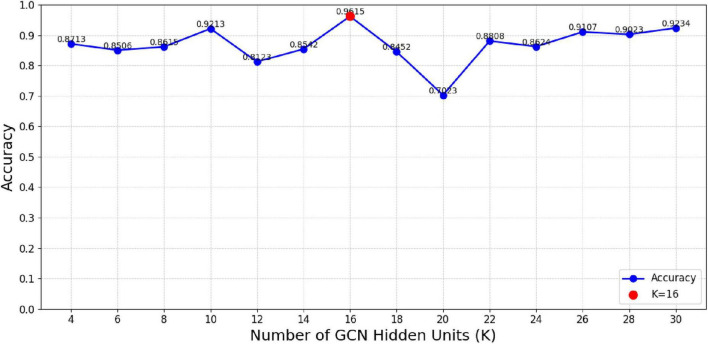
The impact of unit count in the GCN hidden layer (*K*) on classification accuracy.

**FIGURE 9:**
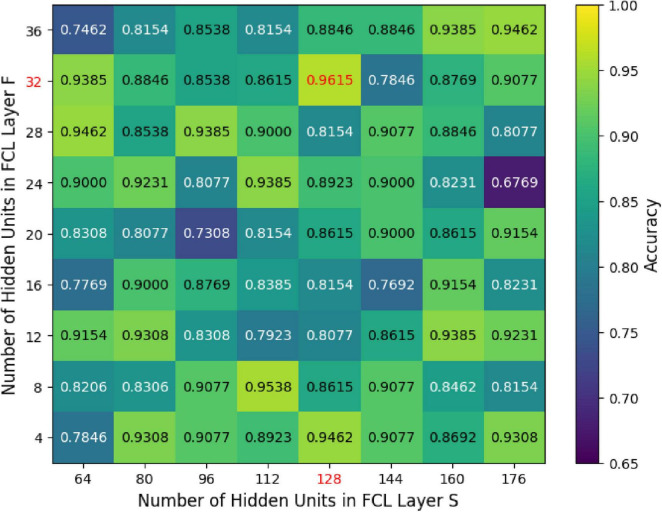
The effect of unit count within the fully connected hidden layers (*S* and *F*) on classification accuracy.

We further investigated how varying the units in the GCN hidden layer (K) and the fully connected hidden layers (S and F) influenced the classification accuracy of the MF-MGCN model. The impact of K, S, and F on accuracy can be observed in Figures 8, 9. Our results indicate that the classification accuracy was highest when K was set to 16, and S and F were set to 128 and 32, respectively. Therefore, in our experiments, we used K = 16, S = 128, and F = 32 as the optimal settings.

### 5.5 Classification effect of different frequency bands

The research further evaluated the MF-MGCN model’s performance across different frequency bands and the full frequency range. The assessment outcomes of the MF-MGCN model for distinguishing AD and HC are presented in Table 5. It is evident that the full frequency band yielded the best results, indicating that multi-frequency analysis enhances the model’s sensitivity to changes in brain activity, thereby improving the accuracy of AD identification. Among the single frequency bands, the Alpha band achieved the highest AUC of 92.36%, demonstrating its significant contribution to the classification outcome.

**TABLE 5:** Classification results of MF-MGCN model for AD and HC across different frequency bands.

Frequency bands	Accuracy (%)	Precision (%)	Recall (%)	F1-score (%)	AUC (%)
Delta	68.46	67.92	60.00	63.72	65.83
Theta	71.54	67.16	75.00	70.87	70.31
Alpha	**88.46**	**83.58**	**93.33**	**88.19**	**92.36**
Beta	68.46	65.08	68.33	66.67	72.07
Gamma	73.08	75.51	61.67	67.89	73.12
Full	**96.15**	**97.67**	**98.33**	**98.00**	**98.74**

This result is consistent with the fact that participants were in a calm state during EEG data acquisition, and the Alpha band is closely associated with relaxation, closed-eye rest, and quiet wakefulness (Lagopoulos et al., 2009). Furthermore, it aligns with previous findings that Alzheimer’s disease patients typically exhibit reduced complexity in the Alpha band and weakened functional connectivity within the default mode network (DMN), both of which are indicative of early neurophysiological deterioration (de Haan et al., 2012b). These observations collectively support the validity and neurobiological relevance of the classification results.

### 5.6 Key EEG channel identification for AD via electrode ablation

To further enhance the interpretability of the model, we conducted a leave-one-electrode-out analysis by sequentially masking each of the 19 EEG electrodes (retaining the remaining 18 in each iteration). For every excluded electrode, we retrained and tested the model, recording the decrease in AUC caused by its removal. Based on the magnitude of AUC reduction, we ranked the importance of each channel to quantify its relative contribution to AD classification.

As shown in Table 6, removing any single electrode resulted in a performance drop to varying degrees, confirming that reducing the number of electrodes negatively affects classification performance. Pz (ΔAUC = 15.27%), F7 (ΔAUC = 12.55%), and F8 (ΔAUC = 11.21%) were identified as the most critical channels for AD classification, underscoring the pathological importance of the parietal and frontal regions in Alzheimer’s disease. In contrast, the removal of electrodes such as O1, Fp2, and Fp1 led to minimal performance degradation (ΔAUC < 2%), indicating their relatively limited contribution to the model’s discriminative power.

**TABLE 6:** Ranking of EEG channel importance derived from leave-one-channel-out analysis.

Channel	Region	AUC (Removed, %)	ΔAUC (%)
Pz	Parietal	83.47	15.27
F7	Frontal	86.19	12.55
F8	Frontal	87.53	11.21
T5	Temporal	88.32	10.42
T6	Temporal	88.67	10.07
C3	Central	89.48	9.26
F4	Frontal	89.73	9.01
P4	Parietal	90.12	8.62
Fz	Frontal	90.55	8.19
C4	Central	90.89	7.85
F3	Frontal	92.37	6.37
T4	Temporal	93.15	5.59
Cz	Central	93.82	4.92
P3	Parietal	94.06	4.68
T3	Temporal	94.33	4.41
O2	Occipital	96.83	1.91
Fp1	Prefrontal	97.12	1.62
Fp2	Prefrontal	97.39	1.35
O1	Occipital	97.67	1.07

## 6 Conclusion and future work

The study introduces an MF-MGCN model for diagnosing AD. The model integrates various EEG data frequency ranges, with each band’s features processed through GCN layers and subsequently combined. We analyzed the functional connectivity strength between different brain electrode channels and conducted experiments on a dataset published in the OpenNeuro repository. The results demonstrated high classification accuracy (96.15%) and AUC (98.74%), effectively detecting AD. The implementation of the MF-MGCN and dataset are available at https://github.com/XQJMJ/MF-MGCN.

Notably, we also evaluated the detection capabilities of the model across various frequency bands. The comparative results indicated that the full-frequency band performed the best, providing more accurate identification of AD and HC. Among individual frequency bands, the Alpha band showed the best performance, with an AUC of 92.36%, demonstrating its significant contribution to the classification outcome. Although the findings emphasize the model’s ability to detect AD, further rigorous testing and validation are required before it can be applied in clinical practice.

While our approach achieved competitive performance, we identified several limitations and potential directions for future improvement. Our dataset was relatively small, which imposed constraints on fitting more complex models. This limitation could lead to instability in larger-scale real-world applications. Future work should validate the generalizability on larger datasets, incorporating data from different racial and regional populations, and conducting research in more diverse cohorts. The current study only utilized EEG signals, future work could explore the integration of other physiological signals (such as MEG, fMRI, etc.) to further improve the model’s detection performance and robustness.

The model proposed in this study is expected to be applied in the initial screening phase of cognitive disorder clinics, assisting doctors in the rapid identification of AD patients. Compared to high-cost imaging methods such as PET and MRI, EEG offers advantages in terms of ease of acquisition, low cost, and strong repeatability, with resting-state EEG taking only about 5 min, making it suitable for large-scale clinical implementation. Additionally, the model, based on the extraction mechanism of multi-frequency band DE values and functional connectivity features, possesses good interpretability. These feature changes can be visually presented using heatmaps or brain network visualization tools, helping clinicians understand the basis for the model’s classification decisions, thus increasing its clinical acceptance.

## Data Availability

The datasets presented in this study can be found in online repositories. The names of the repository/repositories and accession number(s) can be found in the article/supplementary material.
